# Dataset on characterization of recombinant interleukin-23α, IL-12p40 and IL-23 complex protein, which activates JAK-STAT signaling pathway in chicken cell lines using immunocytochemical staining

**DOI:** 10.1016/j.dib.2017.12.008

**Published:** 2017-12-12

**Authors:** Anh Duc Truong, Cong Thanh Hoang, Yeojin Hong, Janggeun Lee, Kyungbaek Lee, Hyun S. Lillehoj, Yeong Ho Hong

**Affiliations:** a Department of Animal Science and Technology, Chung-Ang University, Anseong 17546, Republic of Korea; b Animal Biosciences and Biotechnology Laboratory, Agricultural Research Services, United States Department of Agriculture, Beltsville, MD 20705, USA; c National Institute of Veterinary Research, 86 Truong Chinh, Dong Da, Hanoi, Vietnam

**Keywords:** Chicken, Interleukin-12, Proliferation, Nitric oxide, Signaling pathway

## Abstract

The data herein is related to the research article entitled “Functional analyses of the interaction of chicken interleukin 23 subunit p19 with IL-12 subunit p40 to form the IL-23 complex” [1] where we demonstrated that the chicken interleukin (IL)-23α, IL-12p40, and IL-23 complex regulates Th1, Th17, and Treg cytokine production through heterodimer receptors as well as a homodimer receptor consisting of IL-12Rβ1 and IL-23R, and activates the JAK/STAT signaling pathways. Here, we evaluated the effects of the recombinant chicken IL-23α, IL-12p40, and IL-23 complex protein on cell proliferation and nitric oxide (NO) production in chicken macrophage (HD11) and CU91 T cell lines. In addition, the expression of IL-6, IL-17A, and interferon-γ mRNA were upregulated *in vivo* and *in vitro*. Moreover, treatment with the chicken IL-23α, IL-12p40, and IL-23 complex activated phosphorylation of tyrosine and serine residues in JAK2, STAT1, TYK2, and SOCS1 in chicken cell lines.

**Specifications Table**

**Table:** 

Subject area	*Biology*
More specific subject area	*Chicken interleukin-23, interleukin-12, signaling pathway*
Type of data	*Graph, image, figure*
How data was acquired	*To analyze recombinant proteins, western blot analysis using specific a HRP-anti-His (C-Term) antibody*
	*Nitric oxide content and cell proliferation were measured as described* [2], [3]
	*To analyze signaling protein expression, immunocytochemical analysis using specific bodies were used* [4]
Data format	*Analyzed*
Experimental factors	*Recombinant protein was produced in E. coli and purified using HisPur™ Cobalt Resin; HD11 and OU2 cell lines were treated with recombinant protein as described* [5]
Experimental features	*Analysis of qRT-PCR, western blot, immunocytochemical*
Data source location	*Anseong, Republic of Korea*
Data accessibility	*Data are provided with this article*

**Value of the data**

• The data is valuable for the expression of pro-inflammatory molecules in chicken cell lines treated with DMSO and LPS and various tissues of chicken following *S. enteritidis* infection.
• The data is a contribution to the effect of chicken IL-23α, IL-12p40, IL-12p40 + IL-23α, and IL-23 complex protein to cell proliferation and production of reactive oxygen species in the form of NO in chicken cell lines.
• The data provided the expression of JAK-STAT signaling molecules by chicken IL-23α, IL-12p40, IL-12p40 + IL-23α, and IL-23 complex protein stimulation in chicken cell lines.

## 1 Data

**Fig. 1:**
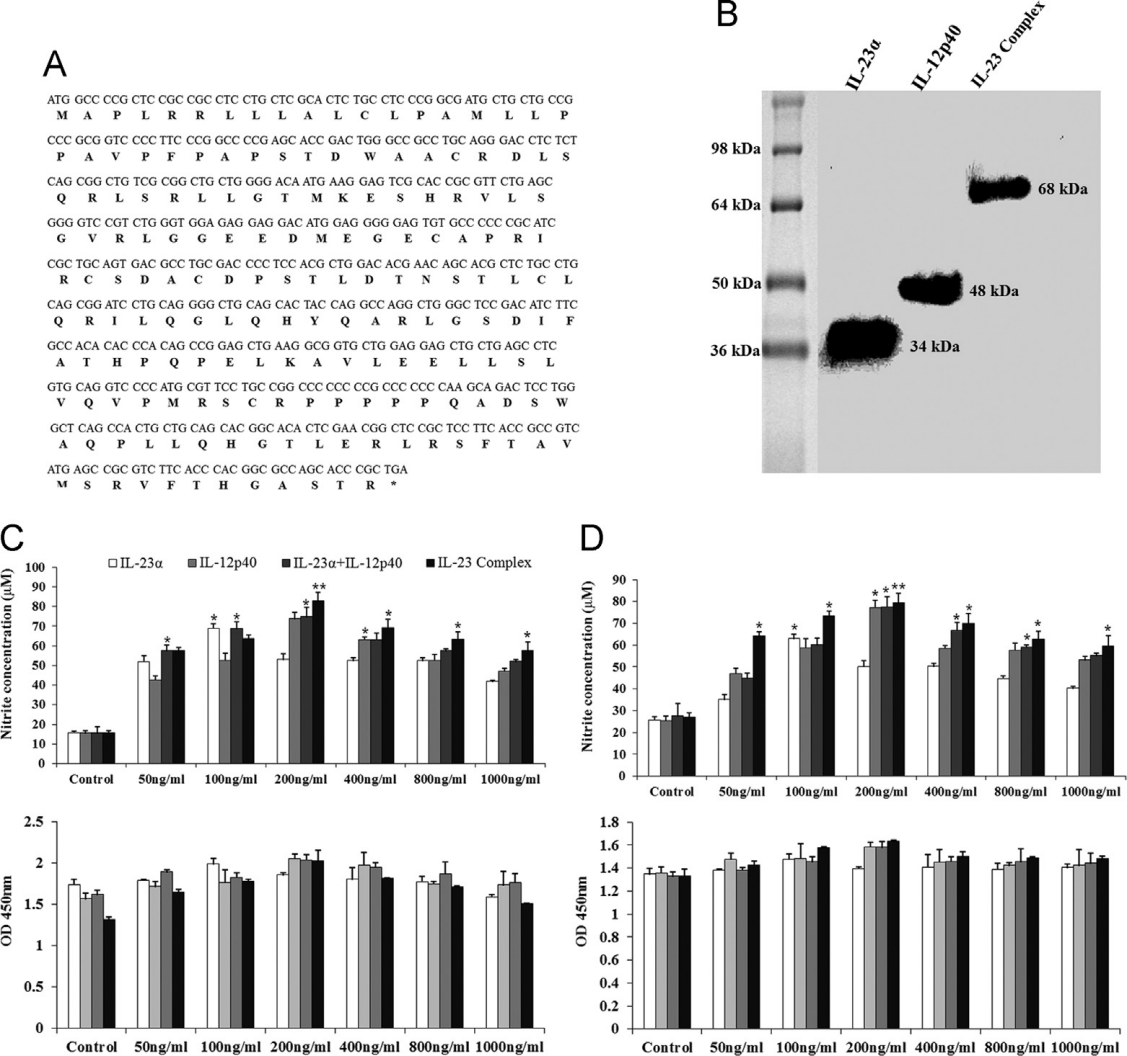
(A) Nucleotide and deduced amino acid sequences of chicken IL-23α. (B) Western blot analysis of chicken IL-12p40, IL-23α, and IL-23 complex recombinant protein using anti-His (C-Term)-HRP antibody. Effect of IL-12p40, IL-23α, and IL-23 complex recombinant protein on cell proliferation and NO production in HD11 cell lines (C) and T cell lines (D). Data (*n*=3) are expressed as the mean±SEM of three independent experiments: *P<0.05 and ** *P*<0.01 vs. control.

**Fig. 2:**
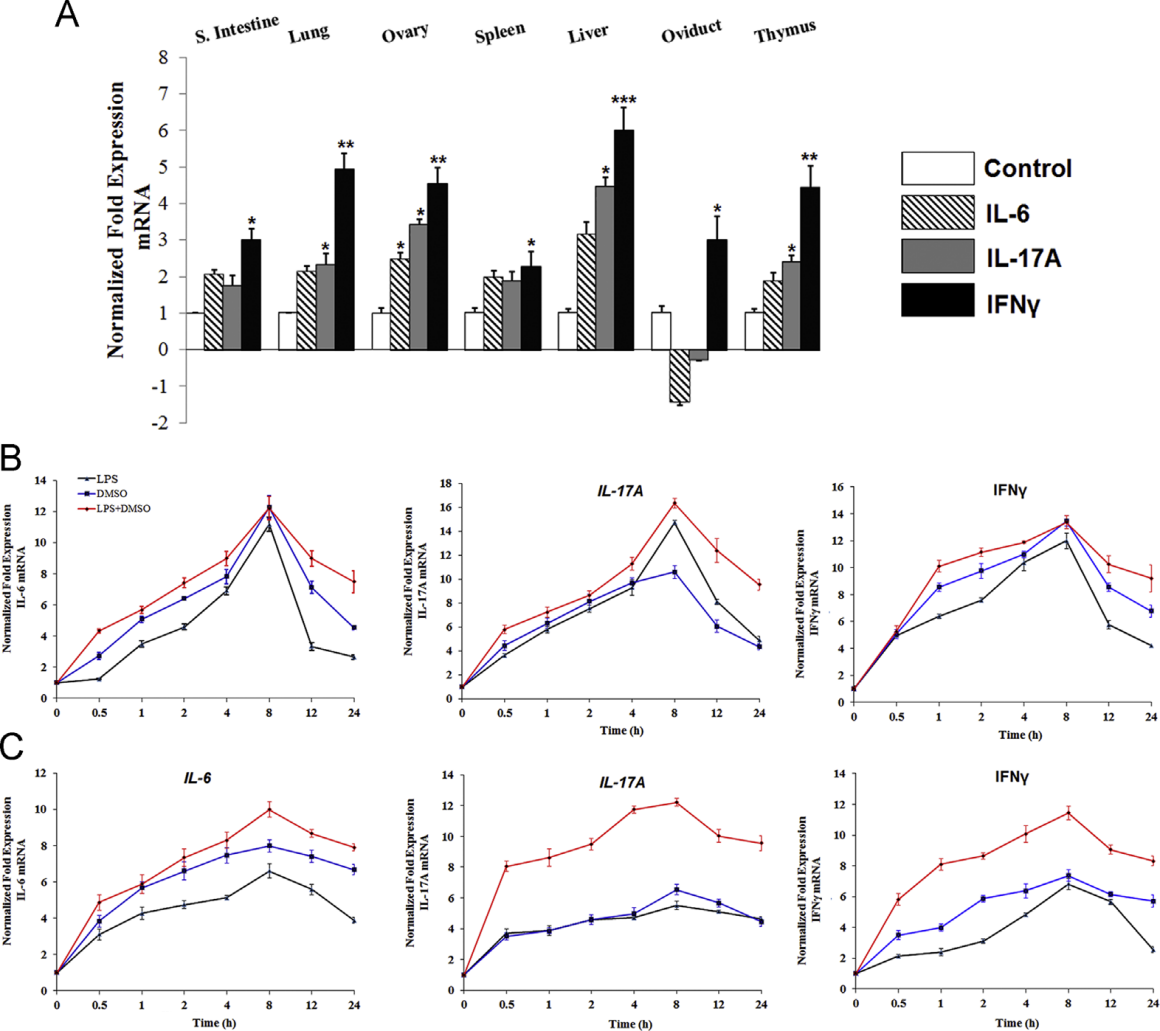
(A) IL-6, IL-17A, and IFNγ mRNA expression in the tissues of chicken infected with 1.0×10^9^ CFU/mL *Salmonella* serovar Enteritidis (S.E.). Tissue samples were collected at 7 days post-infection. Significant differences in mRNA expression levels between treatment to control are indicated as follows: *P<0.05, **P<0.01, and ***P<0.001. Error bars indicate SE (*n*=5) of technical replicates examined in triplicate. Distinct expression of IL-6, IL-17A, and IFNγ in HD11 cell line (B) and CU91 chicken T cell line (C) stimulated with LPS of S.E. (1 µg/mL) and DMSO (1%) for the indicated times. Data are presented as the mean±SEM (*n*=3) of three independent experiments with *P*<0.05.

**Fig. 3:**
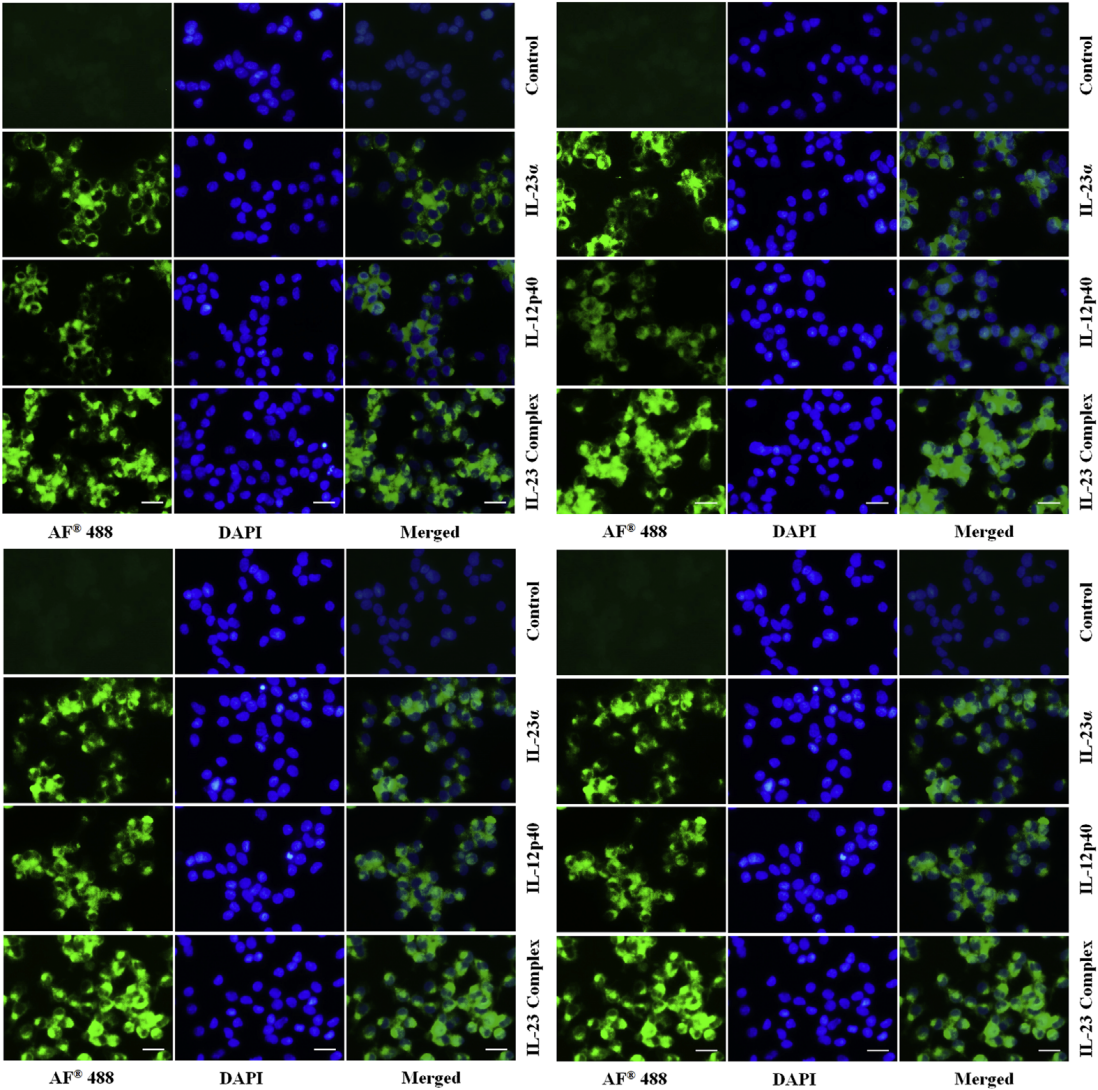
Immunocytochemical analysis of JAK2 (above) and TYK2 (below) signaling proteins in HD11 cell lines (left) and CU91 T cell line (right). Both untreated and recombinant protein treated cells were incubated with primary antibody, Alexa Fluor^®^ 488 Goat Anti-Rabbit IgG (H+L) secondary antibody (green color) and DAPI (blue color) stained. Scale bar 25 µm.

**Fig. 4:**
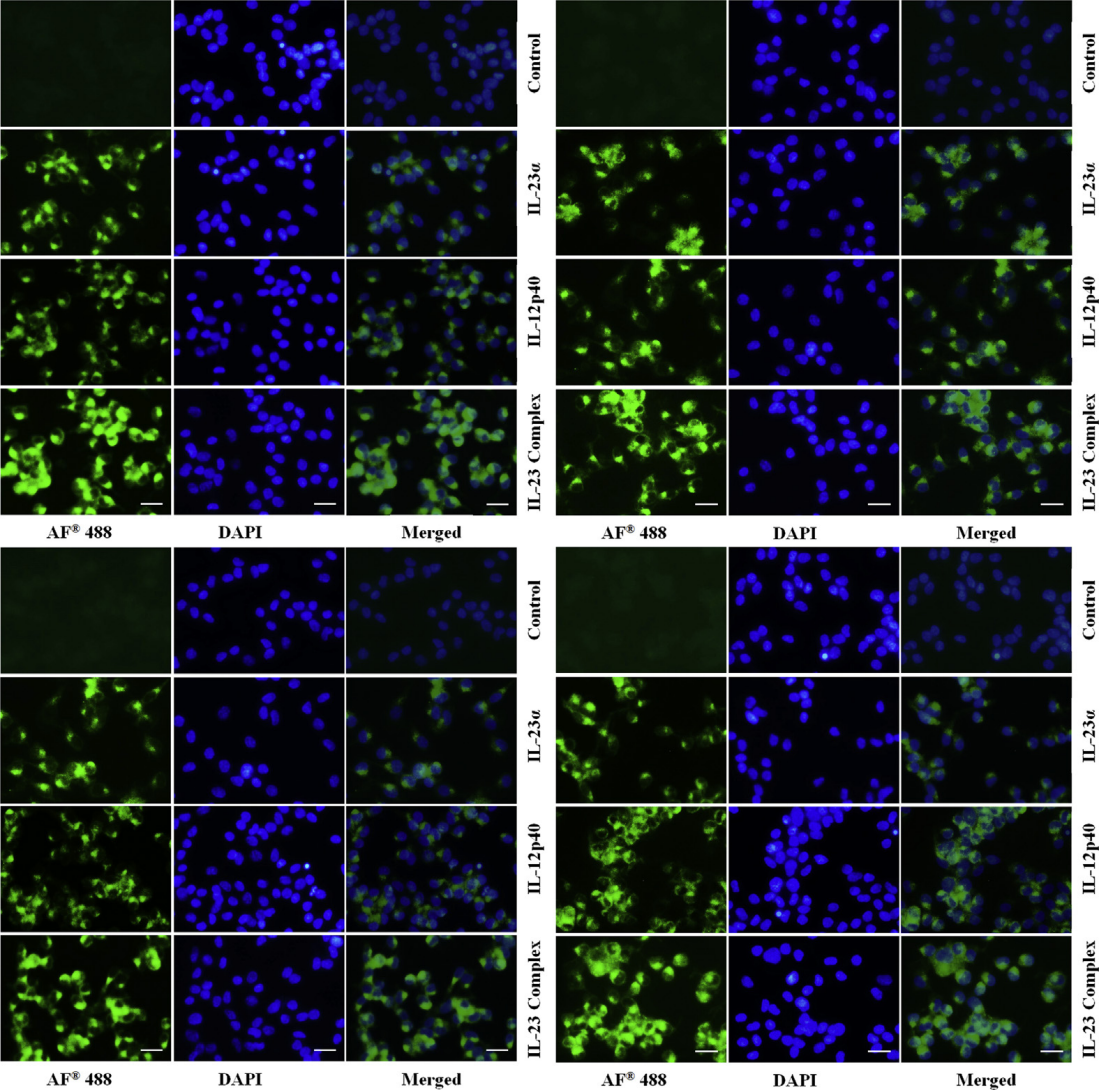
Immunocytochemical analysis of STAT1 (above) and SOCS1 (below) signaling proteins in HD11 cell lines (left) and CU91 T cell line (right). Both untreated and recombinant protein treated cells were incubated with primary antibody, Alexa Fluor^®^ 488 Goat Anti-Rabbit IgG (H+L) secondary antibody (green color) and DAPI (blue color) stained. Scale bar 25 µm.

The dataset in this article provides additional information to Ref. [1], where we demonstrated that the chicken IL-23α, IL-12p40, and IL-23 complex activated multiple signaling pathways through heterodimer receptors, as well as a homodimer receptor consisting of IL-12Rβ1 and IL-23R, and induced Th1, Th17, and Treg cytokine production. In this dataset, we provided the nucleotide and amino acid sequences of the chicken IL-23α coding region (Fig. 1A). These proteins were observed as single bands at 34 kDa (ChIL-23α), 48 kDa (ChIL-12p40- G10S3 linker), and 68 kDa (ChIL-23 complex) (Fig. 1B) by western blotting using the horseradish peroxidase (HRP)-anti-His (C-Term) antibody (Invitrogen, Carlsbad, CA, USA). The size was larger than predicted because of the presence of 3 epitope tags (polyhistidine, S-protein, and thioredoxin) in the recombinant protein. Moreover, data provides information about the effect of chicken IL-23α, IL-12p40, and IL-23 complex protein on cell proliferation and production of reactive oxygen species in the form of NO in both cell lines (Fig. 1C–D). In addition to the dataset, we present the expression of IL-6, IL-17A, and interferon (IFN)-γ mRNA in chicken cell lines treated with dimethyl sulfoxide (DMSO) and lipopolysaccharide (LPS) and in various tissues of the chicken following *Salmonella* Enteritidis infection (Fig. 2). Finally, immunocytochemical analysis showed that the expression of JAK-STAT signaling molecules by IL-23α, IL-12p40, and IL-23 complex stimulation in the chicken cell lines (Fig. 3, Fig. 4).

## 2 Experimental design, materials, and methods

The experimental design is described in details in [1]. Detailed information about the methodology for chicken infection, samples collection, cell stimulation, recombinant protein production and purification, quantitative real-time PCR (qRT-PCR), bioactivity assay, immunocytochemical, western blot and statistical analysis can be found elsewhere [1].
